# Supplementary material for: Experience of personnel involved in dead body management at an apex institute in the aftermath of Odisha triple train collision
Source: PLoS One. 2024 Dec 31;19(12):e0312595. doi: 10.1371/journal.pone.0312595 (PMC11687638; doi:10.1371/journal.pone.0312595)
Supplement: S1 File — (DOCX) [file pone.0312595.s002.docx]

**Experience of faculty and residents in Department of Anatomy in managing the bodies of the deceased in Balasore train accident- June 2023**

**Section 1**

I hereby give consent to participate in this study

**Section 2**

**General Information of the participant:**

Name (optional)

Designation:

Age:

Native of:

**Section 3**

**Qualitative responses:**

a. Write in few sentences about your Personal Experience in Managing the deceased Bodies of Balasore train accident

b. Write a few sentences about the lessons you learnt managing this mass tragedy

c. What are your suggestions to better manage such unprecedented circumstances

d. Have you ever managed such unprecedented situations before?

e. Have you ever managed such unprecedented situation before.

f. What do you think was the strength of team Anatomy managing the deceased bodies of Balasore train accident

g. What do you think was the weakness of team Anatomy managing the deceased bodies of Balasore train accident

**Section 4**

H. Self-reported satisfaction levels of participants during Body Management of Odisha triple train accident June 2023

Please score on a scale of 0-5 how you managed the bodies of Balasore train accident (1= lowest score, 5= highest score)

1. not managed properly

2. just managed

3. managed but could do better

4. well managed

5. very well managed.

A. transport: 1 2 3 4 5

B. photography: 1 2 3 4 5

C. Tagging: 1 2 3 4 5

D. Embalming: 1 2 3 4 5

E. Data Handling: 1 2 3 4 5

F. handing over identified bodies to Police: 1 2 3 4 5

G. Storage: 1 2 3 4 5

I.Grade your work as a team during the train collision:

1. Not Satisfactory
2. Just Satisfactory
3. satisfactory
4. Highly satisfactory
